# Effects of high magnetic field annealing on FePt nanoparticles with shape-anisotropy and element-distribution-anisotropy

**DOI:** 10.1039/d1ra00072a

**Published:** 2021-03-10

**Authors:** Chun Wu, Yanan Jiang, Zhiyuan Niu, Dong Zhao, Wenli Pei, Kai Wang, Qiang Wang

**Affiliations:** School of Materials Science and Engineering, Liaoning Technical University Fuxin 123000 China chun_wu@126.com; Key Laboratory of Anisotropy and Texture of Materials (Ministry of Education), Northeastern University Shenyang 110819 China peiwl@atm.neu.edu.cn; Key Laboratory of Electromagnetic Processing of Materials (Ministry of Education), Northeastern University Shenyang 110819 China

## Abstract

The concave-cube FePt nanoparticles (NPs) with shape-anisotropy and element-distribution-anisotropy were annealed under a high magnetic field (HMF). The NPs underwent spheroidization and phase transformation during the annealing process. The HMF hardly affected the spheroidizing process of NPs, but obviously facilitated the disorder-order transition of the *L*1_0_-phase. The *L*1_0_-phase content, ordering degree, and the coercivity of annealed NPs increased with enhancing the HMF strength. Those results indicated that the nucleation of the *L*1_0_-phase and ordering diffusion of Fe/Pt atoms were promoted by the HMF.

## Abstract 2

The concave-cube FePt nanoparticles (NPs) with shape-anisotropy and element-distribution-anisotropy were annealed under a high magnetic field (HMF).

## Graphical abstract

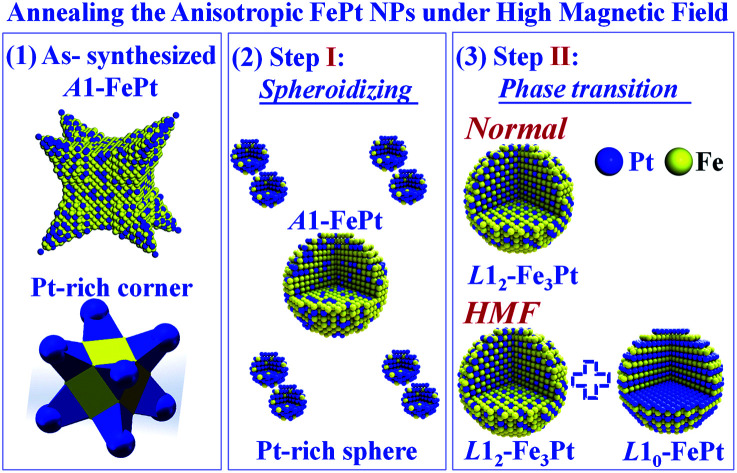

## Introduction

For the wet-chemical synthesized Pt-based binary or multicomponent alloy nanoparticles (NPs), the growth of anisotropic shapes always leads to an anisotropic distribution of the elements.^1,2^ In the Pt–TM (transition metal) alloy NPs, the Pt atom enriches at the corner (along the 〈111〉 direction), and the transition metal atoms prefer to deposit on the surfaces (along the 〈100〉 direction).^1^ The shape anisotropic NPs are well known to become spherical and transform to shape isotropy during the annealing process, however, what happens to the element distribution anisotropy? The wet-chemical synthesized concave-cube FePt NP is a suitable model to solve this puzzle.^3,4^ The shape-anisotropy of concave-cube FePt NPs is originated from the over-growth of truncated-cube or cube seeds along the 〈111〉 direction, and the Pt content is richer at corner-sites, which leads to the element-distribution-anisotropy.^5–7^ Otherwise, the as-synthesized FePt NP always shows a *fcc* disorder structure, and annealing is required to form an *L*1_0_ orderly phase, which has great prospects in the field of magnetic storage, permanent magnets and magnetic catalysis.^8–13^ The typical shape-anisotropy, element-distribution-anisotropy, and the meaningful disorder-order transition of concave-cube *fcc*-FePt NPs offer us an opportunity to study the evolution of microstructure and property for the anisotropic NPs during annealing.

Recently, high-magnetic-field-assisted (HMF-assisted) heat treatment method attracts more and more attentions with the developing of superconducting technology. The morphology of films,^14,15^ growth rates of crystals,^16–19^ and solid-state phase transformation^20–22^ are reported to be regulated by the HMF. For the FePt alloys, the HMF annealing has been reported to induce strain in Fe_3_Pt alloy,^23^ promote disorder-order transformation of FePt nanomaterials,^24–26^ and alignment of FePt spherical NPs.^27–29^ Therefore, if the HMF is applied to anneal the FePt NPs with both the shape-anisotropy and the element-distribution-anisotropy, the influences of the HMF on the NPs in the annealing processes might be clarified.

In this paper, the wet-chemical synthesized concave-cube FePt NPs with shape-anisotropy and element-distribution-anisotropy have been annealed under the HMF, the effects of HMF on shape, size, composition, crystal structure and magnetic properties of the FePt NPs were investigated. It is expected to reveal the influence of the HMF on both the spheroidization and the disorder-order transformation processes.

## Experimental method

The concave-cube FePt NPs were synthesized by a wet-chemical method. Typically, 0.25 mmol Pt(acac)_2_, 25 ml benzyl ether and 0.25 g 1,2-hexadecanediol were mixed, and heated to 105 °C to remove moisture. Surfactants 3 ml OA, 3 ml OAm and precursor Fe(CO)_5_ (0.5 mmol) were injected into the hot solution, and then heated to 220 °C at the rate of 3 °C min^−1^. After refluxing for 60 min, the mixture was naturally cooled to room temperature. Finally, the NPs were washed repeatedly with ethanol, hexane, and dispersed in hexane with concentration about 5 mg ml^−1^. The wet-chemical process was repeated several times to obtain enough FePt NPs.

The annealing process was as follows. About 30 ml of FePt–hexane mixture, 50 ml hexane and 60 g NaCl (ball milling, smaller than 22 μm) were mixed and dried slowly by magnetic stirring at 80 °C. Then, the FePt NPs–NaCl mixture was divided into three parts with equal mass to insure the uniformity of NPs contents during annealing. The as-synthesized FePt NPs were annealed at 700 °C for 1 h in vacuum, which was placed inside a 12 T super-conducting magnet. As the annealing temperature was lower than the melting point of NaCl (803 °C), the NaCl particles acted as a solid insulating medium to prevent agglomeration of NPs during HMF annealing. After annealed, the NPs were collected through centrifuge with water and alcohol, and stored in alcohol at −20 °C.

The shape and size of FePt NPs were characterized by Transmission Electron Microscopy (TEM, JEM-2100F) at an accelerating voltage of 200 kV. Size distributions were collected through counting at least 100 particles in TEM images. The composition of the FePt NPs was analysed by Field Emission Scanning Electronic Microscopy (FE-SEM, SUPRA 35) associated Energy Dispersive Spectroscopy (EDS). The magnetic properties were measured by Vibrating Sample Magnetometer (VSM) at room temperature on a Micro Sense EZ9 magnetometer.

## Results and discussion

**Fig. 1:**
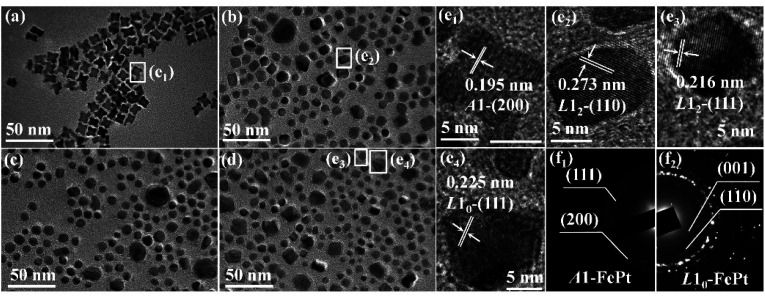
TEM images of (a) as-synthesized and post-annealed FePt nanoparticles at magnetic field of (b) 0 T, (c) 6 T, (d) 12 T. HRTEM images of FePt nanoparticles, (e_1_) from the white box in (a); (e_2_) from the white box in (b); (e_3_) and (e_4_) from the white box in (d). SAED patterns of (f_1_) as-synthesized and (f_2_) 12 T-annealed nanoparticles.

Fig. 1(a) shows the TEM image of as-synthesized FePt NPs, which are in typical concave-cube shapes with a unified size. The TEM images of the samples annealed at 0, 6 and 12 T HMF are showed in Fig. 1(b)–(d), respectively. It can be found that the shape-anisotropic concave-cube NPs transform to sphere after annealing, the size uniformity is poor and some NPs have abnormally grown in the annealed samples. The shapes of cube or decahedron FePt NPs were unchanged after post-annealed.^30,31^ However, the cashew-like and rod-like NPs would transform to sphere after annealing.^32–34^ This means the shape-anisotropic NPs are preferred to become spherical during annealing. Although the NaCl medium is employed to avoid aggregation of the NPs during annealing, the random abnormal growth of the NPs is still unavoidable.^11,35^ As shown in Fig. 1(e_1_)–(e_4_), the lattice fringes of as-synthesized, 0 T-annealed, and 12 T-annealed FePt NPs display interplanar spacing of 0.195, 0.273, 0.216 and 0.225 nm in the particle, which match well respectively with those of the *A*1-(200), *L*1_2_-(110), *L*1_2_-(111) and *L*1_0_-(111) planes. The interplanar spacing of *L*1_0_-(111) is bigger than that of *L*1_2_-(111), which means both *L*1_0_-FePt and *L*1_2_-Fe_3_Pt phases are generated after annealed at 12 T. The SAED patterns of as-synthesized and 12 T-annealed NPs are showed in Fig. 1(f_1_) and (f_2_), respectively. After annealed at 12 T, the diffraction rings of (001), (110) and (002) faces are collected, which also indicates that the *L*1_0_-FePt NPs are generated at 12 T.

**Fig. 2:**
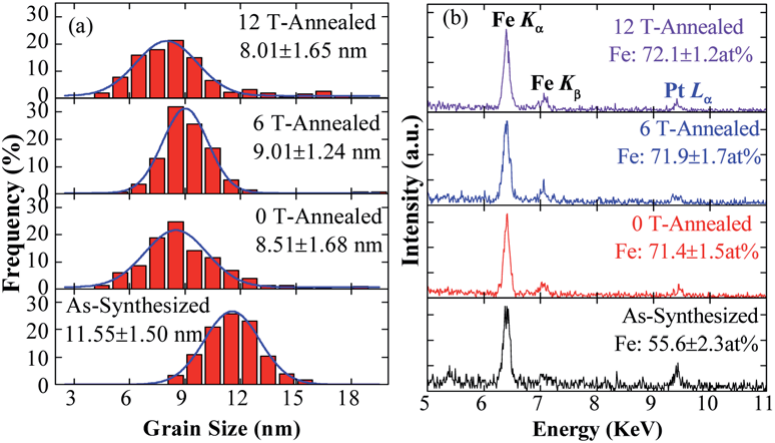
(a) Grain sizes distributions and (b) EDS patterns of as-synthesized and post-annealed FePt nanoparticles.

The grain sizes of the NPs are collected from TEM images, the size distributions are showed in Fig. 2(a). The distributions of the sizes fit well with Gauss function, the average size of the as-synthesized NPs is 11.55 nm, and most of sizes are between 9 and 14 nm. After annealing, the size distributions are moved to left-side, most sizes are between 5 and 11 nm, and the grain size of the FePt NPs decreases to about 8.51 nm. The grain sizes of cashew-like NPs are nearly unchanged,^32^ and the diameters of rod-like NPs are increased after annealed.^33,34^ However, for the concave-cube FePt NPs, the size of concave-cube FePt NPs is decreased. One of the possible reasons is that the bulgy eight corners fell off the concave-cube NP, the over-grew Pt-rich fragments become disconnected from the NPs in the annealing process. When applying the 0, 6 and 12 T HMF during the annealing process, the average size of the NPs remains unchanged within the error range.

The EDS patterns of the NPs are showed in Fig. 2(b). It should be noticed that the relative intensity of Pt *L*_α_ peak decreases after annealing, and the Fe content in the NPs is increased from 51.6% to about 72.1%, which means the Pt atoms in FePt NPs are decreased after annealing. Generally, the composition of the NPs is consistent before and after annealing, because there is no contamination in the NaCl-matrix annealing method and the Fe or Pt will not react with NaCl.^30,31^ Otherwise, the effective interaction depth of EDS (over 10 nm) is bigger than the sizes of NPs, which means all the elements in the NPs can be detected. Thus, the most possible reason for the variation in composition is the roughly collecting or purifying processes. The as-synthesized concave-cube FePt NPs will become spherical, and the Pt-rich corners might fall off from the FePt concave-cubic NPs during annealing. Due to the ultra-small size of the higher Pt-content fragments, it is hard to be collected through the roughly centrifugation. Frankly, the exact reasons why the Pt content decreased after annealing are still unclear, the most possible reason may be that the ultra-small Pt-rich fragments are not fully collected during the roughly centrifugation process.

**Fig. 3:**
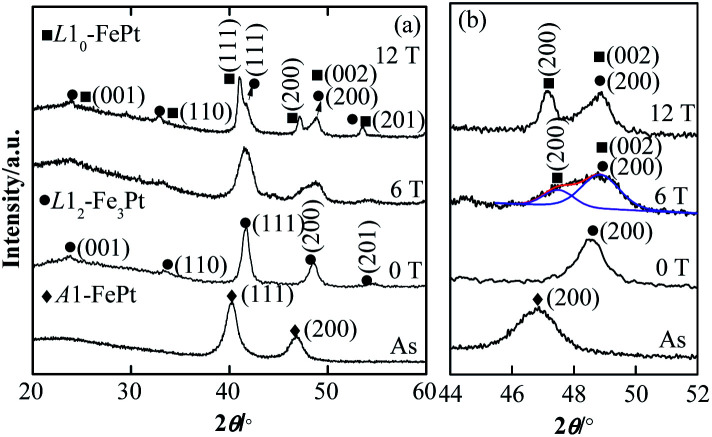
(a) XRD patterns of as-synthesized and post-annealed FePt nanoparticles. (b) Right side shows the enlarge patterns from 44° to 52°.

Fig. 3 shows the XRD patterns of as-synthesized and post-annealed FePt NPs. The as-synthesized FePt NPs is *A*1-FePt phase with a disorder *fcc* structure.^9^ After annealed at 0 T, the diffraction peaks of (001), (110) and (201) faces are detected, which means a *L*1_2_-phase is generated after annealed. The diffraction angles of the (111) faces are one distinguishing feature among *L*1_2_-FePt_3_ (40.452°, PDF#29-0716), *L*1_0_-FePt (41.049°, PDF#43-1359) and *L*1_2_-Fe_3_Pt (41.850°, ref. 36). For the annealed concave-cube FePt NPs, the 41.72° diffraction angle at 0 T more closes to the *L*1_2_-Fe_3_Pt, which consistent with the composition results (closes to Fe_70_Pt_30_) and further illustrates that the Pt content in the NPs is decreased. For the diffraction patterns of samples applied the 6 and 12 T HMF during annealing, a remarkable change is that the (002) peak of *A*1-FePt divides into two peaks, which indicates the formation of *L*1_0_-FePt phase at 6 and 12 T HMF. The separation of (200) and (002) peaks is the most important characteristic of *L*1_0_-FePt, the distance between those two peaks relates to the ordering degree of *L*1_0_-FePt, which suggest that the ordering degree of *L*1_0_-FePt is obviously enhanced at the 12 T HMF.^35,36^ The grain sizes of as-synthesized and post-annealed FePt NPs also calculate by the Scherrer formula from (111) and (002) peaks, the size of as-synthesized NPs is about 8.7 ± 1.1 nm, smaller than the characteristic size from TEM images, which is caused by the concave surface of as-synthesized NPs. After annealed, the grain sizes are calculated to be 10.8 ± 0.6, 10.7 ± 0.5, 11.1 ± 0.8 nm when the HMF strength are 0, 6 and 12 T, receptivity. Those values are bigger than the TEM results, because the annealed magnetic NPs are easy to be aggregated while preparing the XRD-samples.

The ordering degree *s* of the NPs is calculated by *s* = 0.85 × [*I*_(001)_/*I*_(002)_]^0.5^, where *I*_(001)_ and *I*_(002)_ are the intensities of (001) and (002) peaks, respectively. The *s* increases from 0.46 to 0.65 when increasing the HMF from 6 to 12 T, also indicating that the ordering degree can be enhanced by increasing HMF strength. Both the diffraction peaks of *L*1_2_-Fe_3_Pt and *L*1_0_-FePt can be found in the HMF-annealed samples, the content of *L*1_0_-FePt phase in the samples annealed at the 6 and 12 T has been roughly calculated by the *k*-value method. The content of *L*1_0_-FePt phase increases from 16.8% to 25.1%, which means the HMF strength also increases the content of the *L*1_0_-FePt NPs. The crystal structure of the concave-cube *A*1-FePt NPs transforms to *L*1_2_-Fe_3_Pt after annealed, application of the HMF will induce formation of *L*1_0_-FePt, the ordering degree and the content of *L*1_0_-FePt increases with the enhancing of HMF strength.

**Fig. 4:**
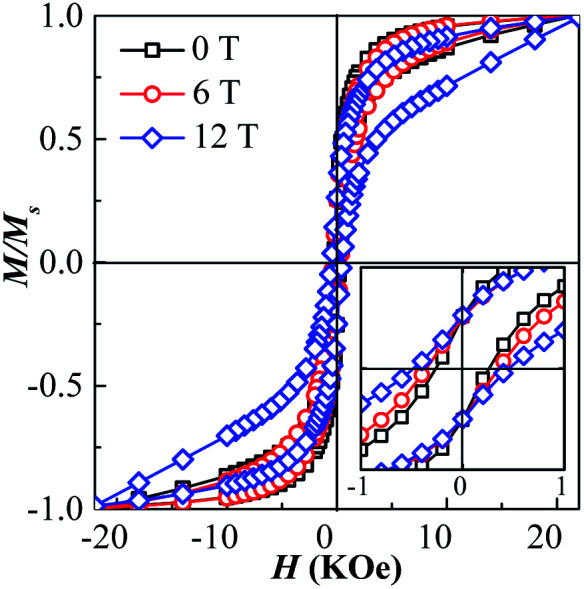
Hysteresis loops of as-synthesized and post-annealed FePt nanoparticles. Inset figure shows the enlarge loops from −1 to 1 kOe.

The room-temperature hysteresis loops of the as-synthesized and the post-annealed FePt NPs are showed in Fig. 4. The nonzero coercivity is detected in the post-annealed FePt NPs, the as-synthesized superparamagnetic FePt NPs show obvious hysteresis behaviour after annealing, because the NPs sizes are bigger than the critical size of *L*1_0_-FePt (2.8 nm) and the *L*1_2_-Fe_3_Pt is the soft magnetic phase. While application of the 0, 6 and 12 T HMF during annealing, the coercivity of the FePt NPs increases from 266 to 363 and 489 Oe, respectively.

The coercivity of the annealed FePt NPs relates to the grain size, effective anisotropy, and phase constitution.^25^ As the grain sizes of NPs are unchanged at 0, 6 and 12 T, the enhancement of coercivity at the 6 and 12 T HMF can be attributed to two reasons, the increasing of ordering degree of *L*1_0_-FePt and the increasing of hard magnetic *L*1_0_-FePt phase content in the annealed samples. However, the coercivity of the sample annealed even at 12 T is not so high, which is due the larger component deviation (far from 1 : 1) with *L*1_0_-phase, the main magnetic phase is the soft magnetic *L*1_2_-Fe_3_Pt phase in the annealed NPs.^25,35,36^

**Fig. 5:**
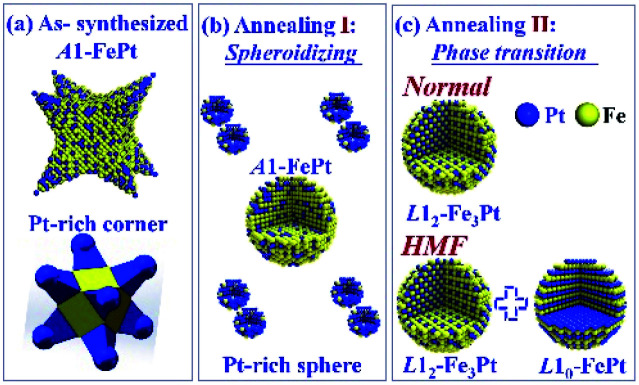
A schematic diagram illustrating the microstructure evolution processes of the concave-cube FePt NP during annealing. (a) The as-synthesized FePt NPs presents a Pt-rich corner. (b) Spheroidizing of the FePt NPs. (c) Phase-transition of FePt NPs at normal condition and under the HMF.

The effects of annealing on the concave-cube FePt NPs are schematized in Fig. 5. The as-synthesized concave-cube FePt are both shape-anisotropic and element-distribution-anisotropic, the Pt atoms rich in the corner-site. In the annealing process (700 °C), the shape-anisotropic FePt NPs become spherical firstly, and then the disorder-order phase-transition occurs. The spheroidization temperature for rod/wire-like, cashew-like, cubic-like NPs are about 400–500 °C, higher than 650 °C, and lower than 700 °C, respectively.^33,34^ For the concave-cube NPs, the spheroidization temperature is less than 700 °C, further speculation, this temperature may be at about 550 °C (close to the critical disorder-order transition temperature), because the size of bulgy corners (about 5 nm) in concave-cube FePt NPs is bigger than nanorods (2–4 nm), and smaller than cashew-like (8–9 nm).^32–34^ Thus, as shown in Fig. 5(b), the concave-cube FePt NPs will divide to eight small Pt-rich spheres and one bigger *A*1-Fe_70_Pt_30_ sphere during annealing. At absence of the HMF, the bigger *A*1-FePt spherical transforms to *L*1_2_-Fe_3_Pt phase but not the *L*1_0_-phase. The possible reasons are as follows: (1) the composition of each wet-chemical synthesized NPs may be not uniform, some Fe-rich NPs will transform to *L*1_2_-Fe_3_Pt phase.^37^ (2) If the Pt atoms diffuse from the in-side to the out-side of the NPs, the *L*1_0_ ordered transition is more favourable. However, the Pt atoms are rich in the out-side (corner) of the concave-cube FePt NPs.^31,32^ (3) With the Pt-rich corner falling off the concave-cube FePt NPs in the spheroidization process, the Fe/Pt atoms ratio in *A*1-FePt spherical closes to 7/3, which is far from 1/1 and benefits to the generation of orderly *L*1_2_-Fe_3_Pt phase.^38^ (4) As the size and mass of the eight small Pt-rich spherical is too small to be centrifugal collected, the Pt content and grain size of the annealed FePt NPs is decreased.

The strength of the HMF cannot affect the shape, size, and composition evolutions of FePt NPs during annealing, but can induce the formation of *L*1_0_-FePt NPs. In the equilibrium phase diagram of FePt alloys, the maximum Fe content in *L*1_0_-phase is about 70%.^36,38^ The Fe content in annealed NPs is about 72.1%, closes to the critical value, which suggests the possibility of formation *L*1_0_-FePt NPs. As the temperature of spheroidization is lower, the Pt-rich corner are fell off still. The disorder-order transition temperature of FePt alloys could be decreased by applying the HMF,^25^ which means that the spheroidization process and the phase-transition process may occur simultaneously under the HMF. This is beneficial to the formation of *L*1_0_ phase during the temperature-raising process at 6 and 12 T. Otherwise, in the phase-transition process (holding at 700 °C), the ordering diffusion of Fe/Pt atoms will be enhanced by the HMF induced magnetization energy,^21–26^ which effect is positively correlated with the HMF strength and leads to the increasing of ordering degree and content of *L*1_0_-FePt phase. However, as the Fe/Pt atoms ratio of the annealed FePt NPs is far from 1/1, only a very small amount of *L*1_0_-FePt NPs with lower orderly degree can be obtained.

## Conclusions

The wet-chemical synthesized concave-cube FePt NPs with shape-anisotropy and element-distribution-anisotropy were annealed under 0, 6 and 12 T HMF. The concave-cube FePt NPs became spherical, the Pt-rich corner-site fell off from the NPs, and the grain sizes were decreased during the annealing. The soft magnetic *L*1_2_-Fe_3_Pt NPs were formed after annealed at 0 T. The HMF didn't influence on the shape, size, and composition of the NPs. The *L*1_0_ ordered FePt NPs were detected in the NPs annealed at 6 and 12 T HMF. The ordering degree, content, and the coercivity of *L*1_0_-FePt NPs increased with enhancing the HMF strength, which suggests that the nucleation of *L*1_0_-phase and ordering diffusion of Fe/Pt atoms were promoted by the HMF.

## Author contributions

Chun Wu: formal analysis, investigation, methodology, writing-original draft and resources. Yanan Jiang, Zhiyuan Niu and Dong Zhao: data curation, formal analysis, investigation and software. Wenli Pei, Kai Wang and Qiang Wang: funding acquisition, project administration, resources, supervision and writing-review & editing.

## Conflicts of interest

There are no conflicts to declare.

## Supplementary Material
